# Untethered Soft Microrobots with Adaptive Logic Gates

**DOI:** 10.1002/advs.202206662

**Published:** 2023-02-21

**Authors:** Zichao Wang, Xuan Zhang, Yang Wang, Ziyi Fang, He Jiang, Qinglin Yang, Xuefeng Zhu, Mingze Liu, Xiaodong Fan, Jie Kong

**Affiliations:** ^1^ MOE Key Laboratory of Materials Physics and Chemistry in Extraordinary Conditions Shaanxi Key Laboratory of Macromolecular Science and Technology School of Chemistry and Chemical Engineering Northwestern Polytechnical University Xi'an 710072 P. R. China

**Keywords:** adaptive logic gates, conjugated logic gates, soft microrobots, stimuli‐responsive hydrogels

## Abstract

Integrating adaptative logic computation directly into soft microrobots is imperative for the next generation of intelligent soft microrobots as well as for the smart materials to move beyond stimulus‐response relationships and toward the intelligent behaviors seen in biological systems. Acquiring adaptivity is coveted for soft microrobots that can adapt to implement different works and respond to different environments either passively or actively through human intervention like biological systems. Here, a novel and simple strategy for constructing untethered soft microrobots based on stimuli‐responsive hydrogels that can switch logic gates according to the surrounding stimuli of environment is introduced. Different basic logic gates and combinational logic gates are integrated into a microrobot via a straightforward method. Importantly, two kinds of soft microrobots with adaptive logic gates are designed and fabricated, which can smartly switch logic operation between AND gate and OR gate under different surrounding environmental stimuli. Furthermore, a same magnetic microrobot with adaptive logic gate is used to capture and release the specified objects through the change of the surrounding environmental stimuli based on AND or OR logic gate. This work contributes an innovative strategy to integrate computation into small‐scale untethered soft robots with adaptive logic gates.

## Graphical abstract

Various soft microrobots with adaptive logic gates and self‐analysis capabilities are designed and fabricated using stimuli‐responsive hydrogels as building modules, which can perform basic logic gates (Yes, AND, or OR gate), connected logic gates (AND‐OR, OR‐OR gates), and intelligently switch logic operation between AND gate and OR gate under varied surrounding environmental stimuli.

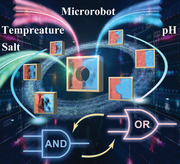

## 1 Introduction

The development of untethered microrobots is one of the holy grails in the field of robotics research. The microrobots have been intensively investigated in the last decade, they can autonomously perform specific and various tasks at a small scale and can be applied to representatively specific environments (e.g., the organism). Meanwhile, they are applied in various fields, such as ecological restoration, biotechnology, micro‐manipulation, targeted release, diagnosis, artificial muscles, and medical imaging.^[^ ^1^, ^2^, ^3^, ^4^, ^5^, ^6^, ^7^, ^8^ ^]^ Intelligent microrobots have reached a high‐level of complexity, which is interacted with the surroundings and can acquire information about the self and the environment and then output the analyzed data as expected.^[^ ^9^, ^10^ ^]^ As a promising strategy, the research of microrobots has currently focused on developing microrobots with advanced stimulus‐responsive abilities, which is a critical step toward micromachine intelligence.^[^ ^11^, ^12^, ^13^ ^]^ Recently, many different kinds of soft microrobots based on responsive polymers have been developed with excellent performances such as programmable shape‐morphing,^[^ ^14^, ^15^ ^]^ drug delivery,^[^ ^16^, ^17^ ^]^ sensors,^[^ ^18^, ^19^ ^]^ and motions (e.g., jumping, swimming and crawling),^[^ ^20^, ^21^, ^22^, ^23^, ^24^, ^25^ ^]^ which provide tremendous potential to achieve intelligent adaptation in diverse fields.

Integrating adaptative logic computation directly into soft microrobots is imperative for the next generation of intelligent soft microrobots as well as for the smart materials to move beyond stimulus‐response relationships and toward the intelligent behaviors seen in biological systems.^[^ ^13^, ^26^, ^27^ ^]^ However, combining adaptive logic‐based computation with untethered soft microrobots is difficult because of the stiffness, flexibility, and size mismatch between rigid electrical components (e.g., electronic chips, control sensors, and batteries) and soft microrobots.^[^ ^9^, ^28^, ^29^, ^30^, ^31^ ^]^ Therefore, it is an enormous challenge to integrate adaptive computing functions into soft microrobots. Combining chemical logic gates with microrobots is a promising strategy to enable microrobots with binary logic computations. Implementing basic logic gates such as YES gate, NOT gate, AND gate, OR gate, and NOR gate is generally supposed to be the precondition for achieving “artificial intelligence” in chemical systems.^[^ ^32^, ^33^ ^]^ Recently, various impressive chemical logic gates performed by chemical compounds or natural molecules have been reported in literature.^[^ ^34^, ^35^, ^36^, ^37^, ^38^, ^39^, ^40^, ^41^ ^]^ Remarkably, Wang and co‐workers further constructed a logic gate based on DNA that could be endowed with adaptiveness, meaning the same logic gate could exhibit two different logic gate functions when the surrounding temperature change.^[^ ^41^ ^]^ Acquiring adaptivity is coveted for soft microrobots that can adapt to implement different works and respond to different environments either passively or actively through human intervention like biological systems. Meanwhile, adaptive logic gates enable the soft microrobots’ flexibility and reusability, which means that the same soft microrobot can implement different logic operations in response to external environmental stimuli without additional replacement.

In this work, a strategy is contributed to fabricate the soft microrobots with logic‐based computation via a simple way and commonly used stimuli‐responsive hydrogels. Stimulus‐responsive hydrogels were widely used for constructing the logic system, which can controllably and reversibly swell and shrink according to external environmental stimuli such as current/voltage, temperature, light intensity, magnetic field, pH, and salt to a controllable and reversible shape transformation.^[^ ^42^, ^43^, ^44^, ^45^ ^]^ Thus, we chose three commonly used stimulus‐responsive hydrogels poly(N‐isopropylacrylamide) (PNIPAm), poly(acrylic acid) (PAAc), poly(2‐acrylamido‐2‐methylpropanesulfonic acid) (PAMPS) as building modules to construct a series of soft microrobots with logic gates, which are occupied by stimuli‐responsive hydrogels except for a hole in the middle. In this logic system, the states of stimuli‐responsive hydrogels (swell or shrink) as input signal, the microrobots can logically and autonomously analyze the input signal to open or close the hole in the middle as the output signal. In this way, the process of logical operation can be observed intuitively. Our work focuses on the proof‐of‐concept demonstration, the soft microrobots fabricated in this work are at the micron or millimeter scale. Our work provides a new approach to integrating adaptive logic gates into soft microrobots so that they can implement two different logic gate operations according to external environmental stimuli without replacement. Furthermore, employing stimuli‐responsive hydrogels as modules to construct soft microrobots with logic gates is a flexible approach. In practical applications, a variety of stimuli‐responsive hydrogels can be selected as modules in response to the different stimuli of the surroundings.

## 2 Results and Discussion

### 2.1 Design and Construction of Soft Microrobots Processing YES, AND, and OR Gates

As a presentation of our notion, a series of submillimeter/millimeter scale microrobots were fabricated including one or more pieces of soft stimuli‐responsive hydrogels with a hole in the middle for capturing objects, and a layer of coating around its sides. (Details on the materials and methods are shown in Supporting Information).

**Figure 1:**
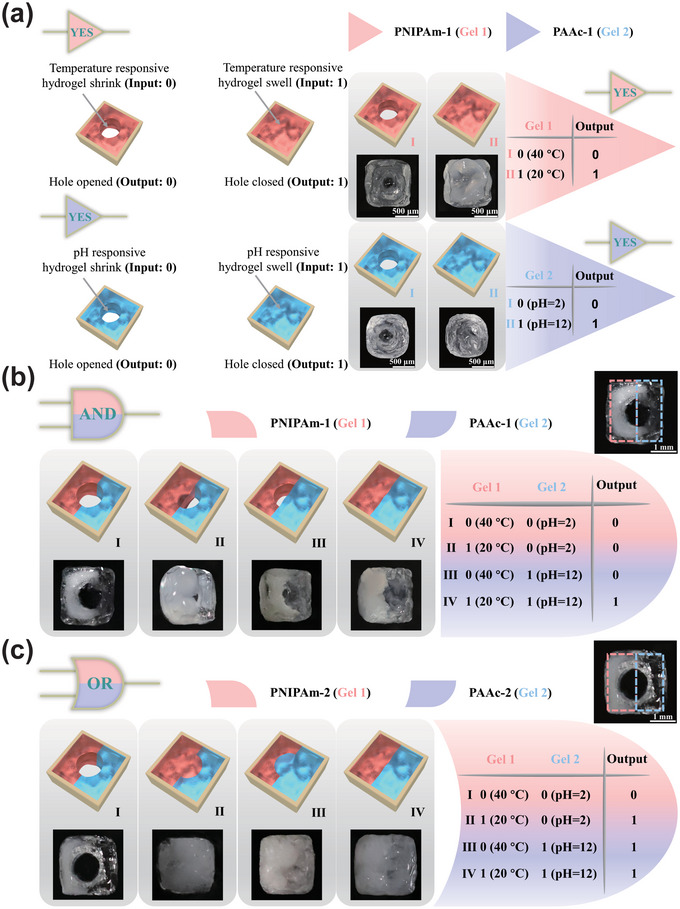
The construction of the microrobots with YES, AND, and OR logic gates employing PNIPAm and PAAc hydrogels. a) Schemes illustrating the microrobots with two YES gates. Inputs 0 and 1 were defined as the state of the hydrogel shrunk and swollen. Outputs 0 and 1 were defined as the state when the hole in the middle of hydrogel opened and closed; Schematic illustrations and images of the microrobots in the different states and the truth tables with b) AND gate and c) OR gate.

In our system, when the hydrogel is in a shrunken state, it is defined as input of 0, when the hydrogel is in a swollen state, it is defined as input of 1. When the hole in the middle of the hydrogel was opened, we define the state as output of 0; when the hole in the middle was fully closed, we define the state as output of 1(**Figure** **1**a). The YES gate was fabricated employing the temperature‐responsive hydrogel (PNIPAm‐1) or pH‐responsive hydrogel (PAAc‐1), respectively (Figure S1, Supporting Information). When the temperature‐responsive hydrogel was placed in an aqueous solution at 40 °C, it was shrunk (input 0). Meanwhile, the hole in the middle of the hydrogel was opened (output 0). Conversely, the hydrogel was swollen at 20 °C (input 1), the hydrogel did not expand laterally outward due to the coating, instead it expanded inward into the hole; hence, the hole was fully closed (output 1). Similarly, the YES gate was performed employing a pH‐responsive hydrogel (Figure 1a). The hydrogel was shrunk in an aqueous solution of pH = 2 (input 0), and the hole in the middle was opened (output 0). By contrast, the hydrogel was swollen in an aqueous solution of pH = 12 (input 1), the hydrogel expanded inward into the hole, and the hole was fully closed (output 1). The process was reversible.

Based on the response properties of PAAc and PNIPAm hydrogel, we constructed more complex logic gates. Figure S2, Supporting Information, demonstrates the typical approaches for constructing the microrobots with AND and OR gates including pH‐responsive and temperature‐responsive hydrogels, each one of them responds to a different stimulus. For the AND gate, two kinds of responsive hydrogels take up half of the microrobot except for a hole in the middle. Half of the microrobot consisting of PAAc‐1 was actuated by pH change, and another half of the microrobot including PNIPAm‐1 was actuated by temperature change. When the temperature decreases or the pH increases, the half of microrobot was expanded to the center. However, it merely took up half of the hole without fully closing the hole. Hence, the hole could be fully closed only when both the hydrogels expanded (Figure 1b). For the OR gate, the two stimuli‐responsive hydrogels (PAAc‐2 and PNIPAm‐2) occupy half of the microrobot except for a hole in the middle. Unlike the AND gate microrobot, whenever anyone (or both) hydrogel of OR gate microrobot was swollen under the influence of either one (or both) of the stimulus, the hole was closed to zero (Figure 1c).

### 2.2 Design and Construction of Soft Microrobots Processing Combinational Logic Gates

**Figure 2:**
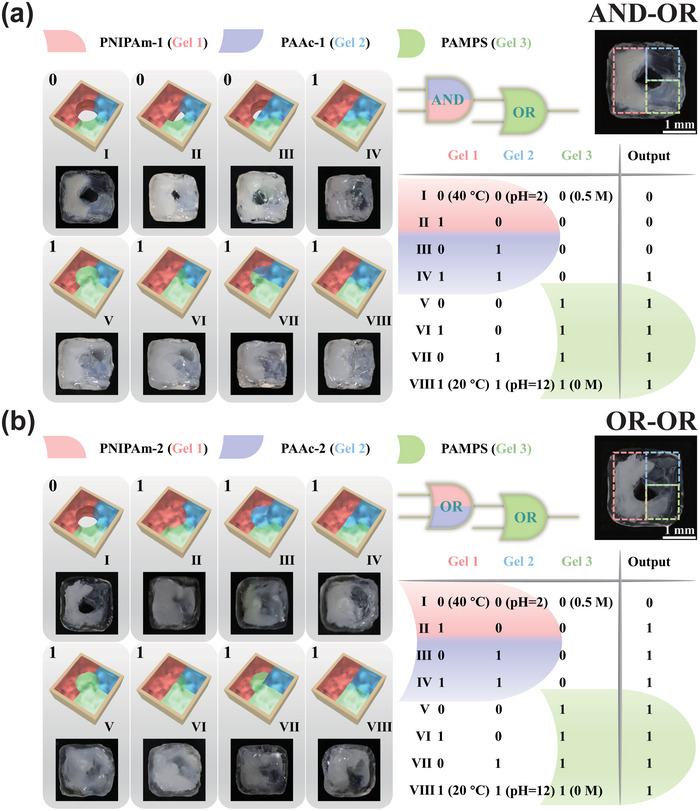
Schematic illustrations and images of the microrobots in the different states with connected logic gates and the truth tables. a) An AND gate connected with an OR gate. b) An OR gate connected with another OR gate.

Most chemical logic gates can only exhibit basic logic functions, which limits their applications for performing more advanced operations. Connecting different basic chemical logic gates can construct multi‐switchable logical systems and be applied in various fields, such as molecular computation,^[^ ^36^, ^46^ ^]^ chemical detection,^[^ ^47^, ^48^ ^]^ and molecular capturing and releasing.^[^ ^49^, ^50^ ^]^ Reasonably advanced arithmetic operations can be performed by integrating basic logic gates. In our work, different classes of stimuli‐responsive hydrogels were employed to construct soft microrobots with combinational logic gates. Under different types of external stimuli, the outputs of the microrobot with AND and OR gate are the same: occupying space of the hole in the middle by expansion or contraction. Thus, we can integrate different basic logic gates to implement more advanced computations. First, we integrated AND gate and OR gate into the same microrobot, which was taken up by three kinds of responsive hydrogels except for a hole in the middle (Figure S3, Supporting Information). The AND gate was implemented including a temperature‐responsive hydrogel (PNIPAm‐1) and a pH‐responsive hydrogel (PAAc‐1) as discussed in Figure 1b. The OR gate was fabricated employing a salt‐responsive hydrogel poly(2‐acrylamido‐2‐methylpropane sulfonic acid) (PAMPS). When both the hydrogels (PNIPAm‐1 and PAAc‐1) of the AND gate were swollen, the hole in the middle was fully closed regardless of whether the hydrogel (PAMPS) was swollen (in aqueous solution) or shrunk (in 0.5 m NaCl solution). However, when the hydrogel (PAMPS) was swollen, the hole was fully covered regardless of whether the hydrogels (PNIPAm‐1 and PAAc‐1) of the AND were swollen or not (**Figure** **2**a). Moreover, we also fabricate a soft microrobot that connected an OR gate to an OR gate.

The first OR gate was implemented including hydrogels (PNIPAm‐2 and PAAc‐2) as discussed in Figure 1c. The second OR gate was also fabricated by salt‐responsive hydrogel (PAMPS). The preparation method was consistent with that of microrobot with AND‐OR gate. Whenever anyone (or both) hydrogel of the first OR gate was swollen, the hole in the middle was fully closed regardless of whether the hydrogel (PAMPS) was swollen or shrunk. Likewise, when the hydrogel (PAMPS) was swollen, the hole was also fully covered regardless whether the hydrogels (PNIPAm‐1 and PAAc‐1) of the OR gate were swollen or not (Figure 2b).

The reversibility of the microrobots that performed logic gates operation was tested by switching the different types of stimuli repeatedly for ten cycles. For the microrobot performing YES gate by pH‐responsive hydrogel, we changed the pH of the solution repeatedly from pH 2 to pH 12 and vice versa (Figure S4a, Supporting Information); For the microrobot performing YES gate by temperature‐responsive hydrogel, we changed the temperature repeatedly from 40 to 20 °C and vice versa (Figure S4b, Supporting Information). In both cases, the response times of the microrobots performing logic gates remained approximately constant throughout the ten cycles. These results show that the microrobots were able to operate logic gates without any decrease in performance after ten cycles of changing the stimuli. This conclusion is supported by previous studies in which the stimuli‐responsive hydrogels were known to be able to change their sizes reversibly for many cycles.^[^ ^51^ ^]^

### 2.3 Design and Construction of Soft Microrobots with Adaptive Logic Gates

**Figure 3:**
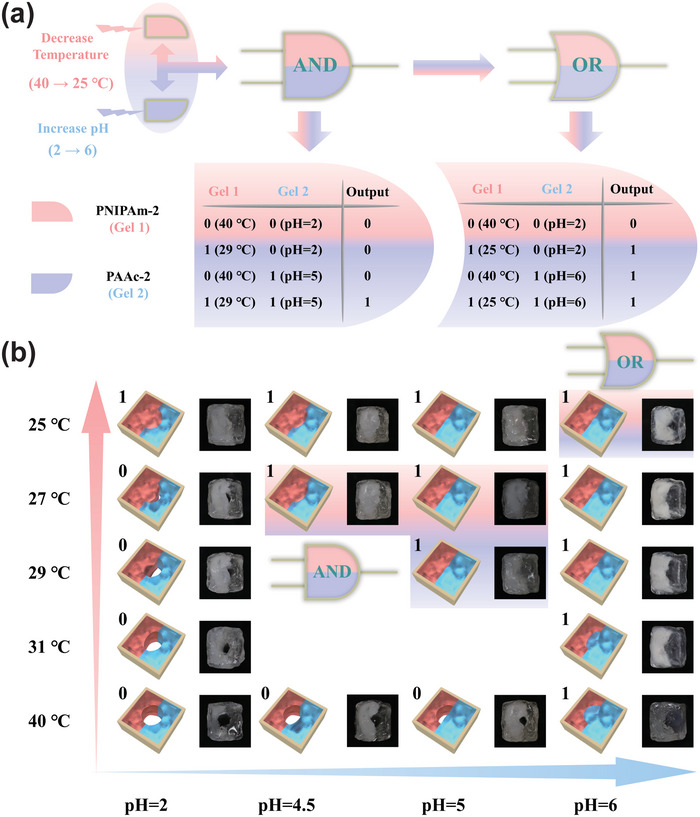
The construction of soft microrobots with adaptive logic gate. a) The schematic diagram to switch logic operations between OR gate and AND gate and truth tables. b) Schematic illustrations and images of the soft microrobots switching AND gate to OR gate.

More importantly, many intelligent biological systems such as humans, animals, and even cells can choose appropriate logic operations to adapt to surrounding changes. For example, the lactose operon is only activated for the production of monosaccharides in the presence of lactose and interestingly, when both glucose and lactose exist, the lactose operon is inactivated. Therefore, the energy needed by cells was preferred to provide by glucose instead of the less efficient lactose.^[^ ^40^, ^52^ ^]^ Although the soft microrobots we fabricated can perform a series of logic operations (e.g., YES, OR, AND, and connected gates) upon the environmental stimuli, they are not able to choose logic gate intelligently under different situations since one microrobot can only perform one logic gate. Hence, we investigate the effect of amount of crosslinkers on the swell ratio of stimuli‐responsive hydrogels, and adjust the swell ratio of hydrogels to develop the microrobot that can smartly switch logic operation between AND gate and OR gate upon varied environmental stimuli (**Figure** **3**a). The microrobot was designed based on OR and AND gates. At first, the microrobot was placed in an aqueous solution of pH = 2 at 40 °C, it was in a fully shrunk state. As the surrounding environmental conditions change, when the temperature was between 27 and 29 °C, and the pH was between 4.5 and 5, only one type of responsive hydrogel of the microrobot was swollen, the hole in the middle cannot be fully closed (output 0). When two kinds of stimuli‐responsive hydrogel were swollen simultaneously, the hole in the middle was fully closed (output 1). The microrobot could identify this environmental information and perform the AND gate. However, when the temperature was lower than 25 °C and the pH was higher than 6, any one (or both) type of responsive hydrogel of microrobot was swollen, the hole in the middle of the microrobot was able to fully close (output 1). The microrobot could analyze the environmental stimuli and switch to the OR gate intelligently (Figure 3b).

**Figure 4:**
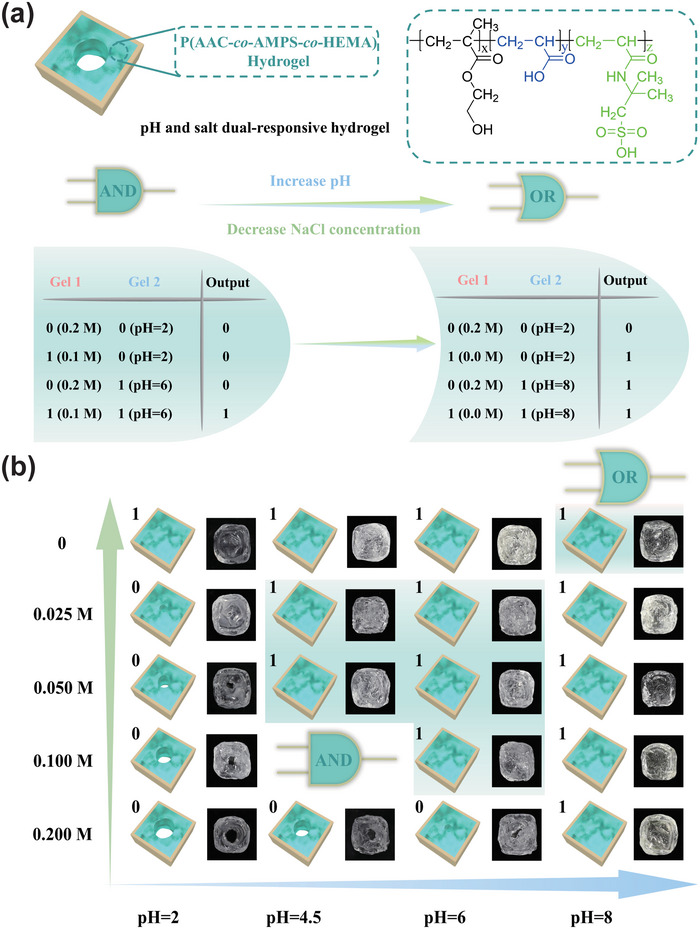
The construction of soft microrobots with adaptive logic gate employing the pH and salt dual‐responsive hydrogel. a) The schematic diagram to switch logic operation between AND and OR gate and truth tables. b) Schematic illustrations and images of the soft microrobots switching AND gate to OR gate.

The soft microrobot with adaptive logic gates demonstrated in Figure 3 was constructed by two independent stimulus‐response hydrogels (PNIPAm and PAAc), which contributes an advantage that autocephalous stimulus‐response modules were not affected by different stimulus signals. In order to show the universality of constructing the microrobots with adaptive logic gates, we introduce a more convenient strategy for developing the soft microrobot with adaptive logic gates employing the dual‐responsive hydrogel, which was prepared by pH‐responsive polymer (PAAc) and salt‐responsive polymer poly(2‐acrylamido‐2‐methylpropane sulfonic acid) (PAMPS) as shown in **Figure** **4**a. The construction method is the same as the YES gate (Figure S5, Supporting Information). The microrobot also demonstrated the characterization of switching between AND and OR logic gates according to different NaCl concentrations and pH of the aqueous solution. In the same way, when the hydrogel is in a shrunken state, it is defined as an input of 0, when the hydrogel is in a swollen state, it is defined as an input of 1. In addition, the PAAc network and the PAMPS network of the hydrogel are labeled as Gel 1 and Gel 2, respectively.

At first, the microrobot was exposed to a 0.2 m NaCl aqueous solution of pH = 2, it was in a fully shrunk state. As the surrounding environmental conditions change, when the concentration of NaCl solution was between 0.025 and 0.1 m, and the pH was between 4.5 and 6, the soft microrobot could identify this environmental information and perform the AND gate. However, when the concentration of NaCl solution was 0 m and the pH was higher than 8, the soft microrobot could analyze the environmental stimuli and switch to the OR gate intelligently (Figure 4b). The process of the microrobot performing OR gate in an aqueous solution of pH = 8 is shown in Figure S6, Supporting Information.

### 2.4 Magnetic Soft Microrobot with Adaptive Logic Gate Performing Capture, Delivery, and Release

**Figure 5:**
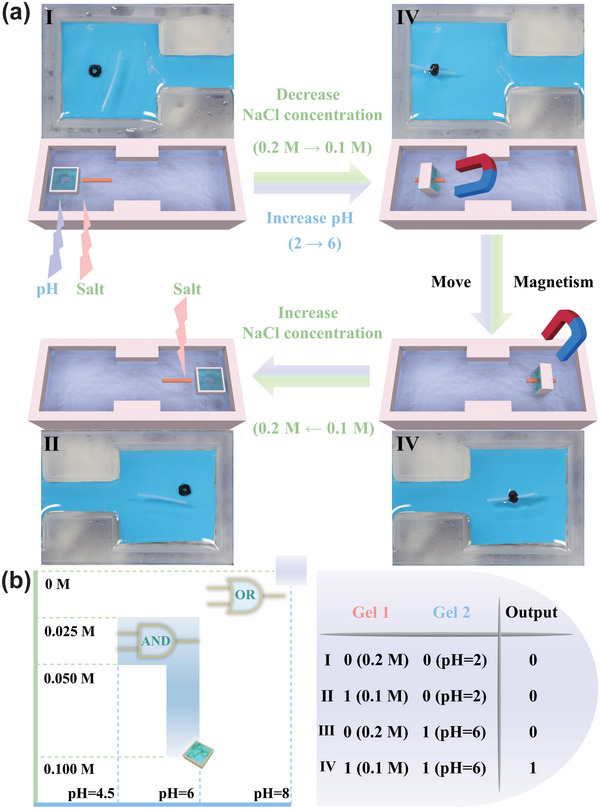
a) Schematic illustrations and images of the process that a Teflon rod was transferred by the magnetic soft microrobot with adaptive logic gates based on an AND gate. b) The area of the AND gate and truth tables.

**Figure 6:**
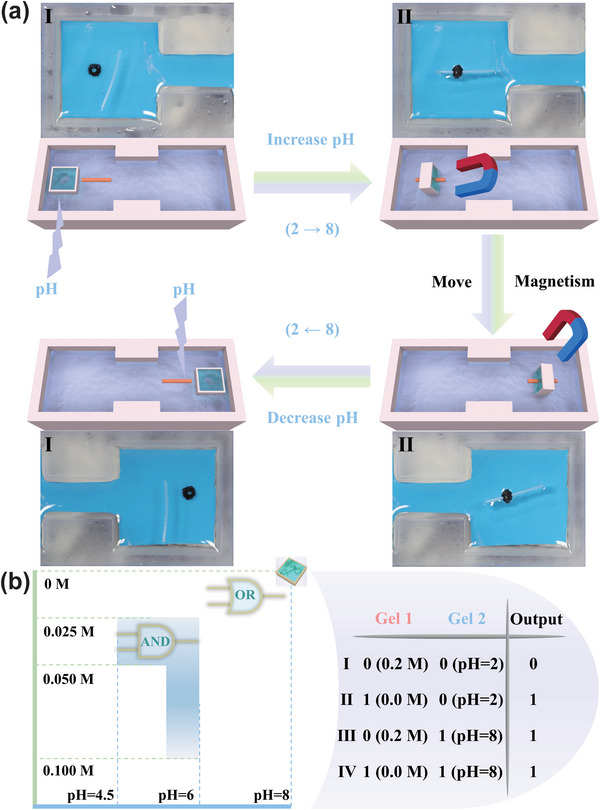
a) Schematic illustrations and images of the process that a Teflon rod was transferred by the magnetic soft microrobot with adaptive logic gates based on an OR gate. b) The area of the OR gate and truth tables.

One important application of these microrobots is that they could be used for the targeted transport of therapeutic agents. As a proof of concept, we demonstrated the trapping, delivery, and release of a Teflon rod (diameter: 200 µm) using a pH and salt‐actuated microrobot controlled under tele‐operation. By adding iron oxide (Fe_3_O_4_) nanoparticle to the dual‐responsive hydrogel as shown in Figure 4a, the soft microrobots are endowed with magnetic properties. Under the action of the magnetic field, the microrobot can reach the designated position. We simulated the changes in environmental conditions and chose the pH and salt concentration that the soft microrobot can perform the logic operation based on AND gate or OR gate as shown in Figure 4b. First, the microrobot was guided to the Teflon rod using a pulling motion at a 0.2 m NaCl aqueous solution of pH = 2. As the microrobot captured the Teflon rod and added the aqueous solution of pH = 6, the hole in the middle began to close. When the concentration of NaCl aqueous solution was reduced to 0.1 m, the hole in the middle fully closed and the Teflon rod was gripped tightly by the microrobot. Then, the trapped Teflon rod was then freely moved along the desired path using a swimming motion. Finally, with the addition of high‐concentration NaCl solution, the hole began to open, releasing the trapped Teflon rod. The process is based on an AND gate (**Figure** **5**). Similarly, the Teflon rod can be captured and released by the same microrobot based on an OR gate just by changing the pH of the solution (**Figure** **6**). Hence, the soft microrobot could analyze the environmental stimuli and choose the logic gate intelligently.

## 3 Conclusion

In summary, this work introduced a strategy to construct a series of small‐scale untethered soft microrobots with adaptive logic gates based on stimuli‐responsive hydrogels. The various stimuli‐responsive hydrogels (i.e., temperature, pH, and salt‐responsive hydrogels) were used as modules to fabricate soft microrobots performing different logic gates (i.e., YES, AND, and OR gate). Besides, we successfully integrated different basic logic gates into the one soft microrobot as described (i.e., AND‐OR and OR‐OR gate). Most importantly, we developed two kinds of soft microrobots that can adapt logic operations between AND gate and OR gate according to the surrounding environmental stimuli intelligently and autonomously. Thus, combined with the simplicity of preparation and the capability of the adaptive logic gates, the soft microrobots own anticipated potential to perform advanced computation or analyses. The proposed strategy for fabricating the microrobots is able to build a soft adaptive computational system directly into the body of the microrobots. This would lead to a new generation of soft microrobots, paving the way for more sophisticated soft microrobots and intelligent compliant structures.

## Conflict of Interest

The authors declare no conflict of interest.

## Supporting information

Supporting Information

## Data Availability

The data that support the findings of this study are available from the corresponding author upon reasonable request.
