# Supplementary material for: Untethered Soft Microrobots with Adaptive Logic Gates
Source: Adv Sci (Weinh). 2023 Feb 21;10(13):2206662. doi: 10.1002/advs.202206662 (PMC10161047; doi:10.1002/advs.202206662)
Supplement: Supplementary file 1 — Supporting Information [file ADVS-10-2206662-s001.pdf]

ADVANCED SCIENCE
Open Access

Supporting Information

for *Adv. Sci.*, DOI 10.1002/advs.202206662

Untethered Soft Microrobots with Adaptive Logic Gates

Zichao Wang, Xuan Zhang, Yang Wang, Ziyi Fang, He Jiang, Qinglin Yang, Xuefeng Zhu, Mingze Liu, Xiaodong Fan and Jie Kong**

Untethered soft microrobots with adaptive logic gates

Zichao Wang[#], Xuan Zhang*[#], Yang Wang, Ziyi Fang, He Jiang, Qinglin Yang,

Xuefeng Zhu, Mingze Liu, Xiaodong Fan and Jie Kong*

MOE Key Laboratory of Materials Physics and Chemistry in Extraordinary Conditions, Shaanxi Key Laboratory of Macromolecular Science and Technology, School of Chemistry and Chemical Engineering, Northwestern Polytechnical University, Xi'an, 710072, P. R. China.

*Corresponding Author, E-mail: zhangxuan@nwpu.edu.cn; kongjie@nwpu.edu.cn

[#]These authors contributed equally to this work

Materials

N-isopropylacrylamide (NIPAm, >98.0%), Acrylic acid (AAc, >99.0%), 2-hydroxyethyl methacrylate (HEMA, >95.0%), acrylamide (AAm, >98.0%), 2-acrylamido-2-methylpropanesulfonic acid (AMPS, >98.0%) were purchased from TCI company. Ethylene glycol dimethacrylate (EGDMA, 98%), N, N'-methylenebis (acrylamide) (MBAA, 99%), 2,2′-Azobis (2-methylpropionamidine) Dihydrochloride (V50, 97%) were purchased from Sigma-Aldrich. Ammonium persulfate (APS), ethanol, sodium chloride (NaCl) were purchased from Aladdin Reagent Co., Ltd., China. Iron (II, III) oxide particles (Fe_3O_4, 20 nm diameter) was purchased from Innochem company. Silicon elastomer (Ecoflex 00-30) was purchased from Smooth-On.

Fabricating the YES gate.

The soft microrobot with YES gate was prepared using the precursor solution for the temperature-responsive hydrogel (PNIPAm-1) was composed of NIPAm 373 mg, MBAA 5.4 mg, V50 7.75 mg, DI water 1.43 g and for the pH responsive hydrogel (PAAc-1) was composed of AAc 0.04 g, HEMA 0.36 g, MBAA 3 mg and V50 4 mg, DI water 1.6 g. For varying the thickness of responsive layer and passive layer, appropriate Ecoflex mold were selected. First, a kind of plastic mold (1 mm × 1mm × 0.5 mm) with a rod in the center (diameter: 500 μm) were fabricated using a printed plastic mold by a 3D printer as shown in **Figure** S1a. The Ecoflex mold was obtained by molding as shown in Figure S1b. The precursor solution for the stimuli-responsive hydrogel was introduced into the Ecoflex mold with through injector. This mixture was

polymerized under UV irradiation for 10 min. Next, the hydrogel was immersed into water overnight to remove the unreacted monomer and initiator. After that, the four sides of the hydrogel were fixed with glue to obtain the final soft microrobot with the PAAc (the thickness of glue: 100 μm), and another soft microrobot with the PNIPAm (the thickness of glue: 100 μm), as shown in Figure S1d.

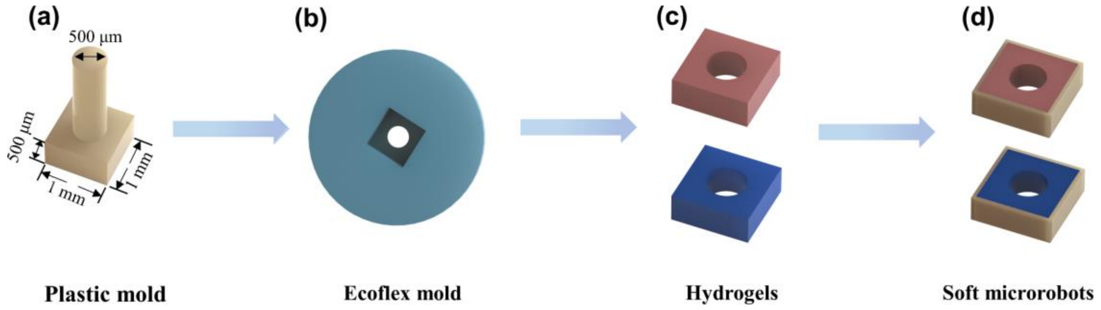

Figure S1. The preparation of soft microrobot with YES gate.

Fabricating the AND and OR gate.

The soft microrobots with AND gate was prepared using the precursor solution for the temperature-responsive hydrogel (PNIPAm-1) was composed of NIPAm 373 mg, MBAA 5.4 mg, V50 7.75 mg, DI water 1.43 g. The precursor solution for the pH responsive hydrogel (PAAc-1) was composed of AAc 0.04 g, HEMA 0.36 g, MBAA 3 mg and V50 4 mg, DI water 1.6 g. The soft microrobots with OR gate was prepared using the precursor solution for the temperature-responsive hydrogel (PNIPAm-2) was composed of NIPAm 373 mg, MBAA 2.7 mg, V50 7.75 mg, DI water 1.43 g. The precursor solution for the pH responsive hydrogel (PAAc-2) was composed of AAc 0.04 g, HEMA 0.36 g, MBAA 1.5 mg and V50 4 mg, DI water 1.6 g. As shown in

Figure S2a, the hydrogel soft microrobot with environmentally responsive logic gates were prepared using a micromolding technique. First, two kinds of plastic mold with different sizes were fabricated using a printed plastic mold by a 3D printer. These plastic molds were fixed in a petri dish by glue. Then, a mixture of Ecoflex prepolymer and its curing agent (1:1 ratio) was prepared as the material for the Ecoflex micromold. Then, the mixture was poured over a 3D printed plastic mold and cured at 65 °C. Finally, the Ecoflex mold with a hole in the center was obtained after peeled off the 3D printed plastic master (Figure S2b). The thickness of the hydrogel layer and the diameter of the hole were determined by the depth of the Ecoflex mold, which can be controlled by varying the size of the 3D printed plastic mold. Next, a PTFE rod was used to plug the hole in the Ecoflex mold with different sizes. The ultraviolet (UV)-curable precursor solution for the pH-responsive hydrogel was introduced into the Ecoflex mold with small size through injector. This mixture was polymerized under UV irradiation for 20 min (at a wavelength of 365 nm and an intensity of 60 mW cm^{-2}). Then, the cured pH-responsive hydrogel was transferred into the Ecoflex mold with large size and the precursor solution for the temperature-responsive hydrogel was introduced into the same Ecoflex mold. This mixture was polymerized under UV irradiation for another 20 min (Figure S2c). After removing the PTFE rod, the half temperature responsive and half pH responsive hydrogel (with a hole in the center) was extracted from the mold and washed in DI water overnight to remove the unreacted monomer and initiator. Finally, the four sides of the hydrogel were fixed with glue to obtain the final soft microrobot with AND and OR gate (Figure S2d) (the thickness of glue: 200 μm).

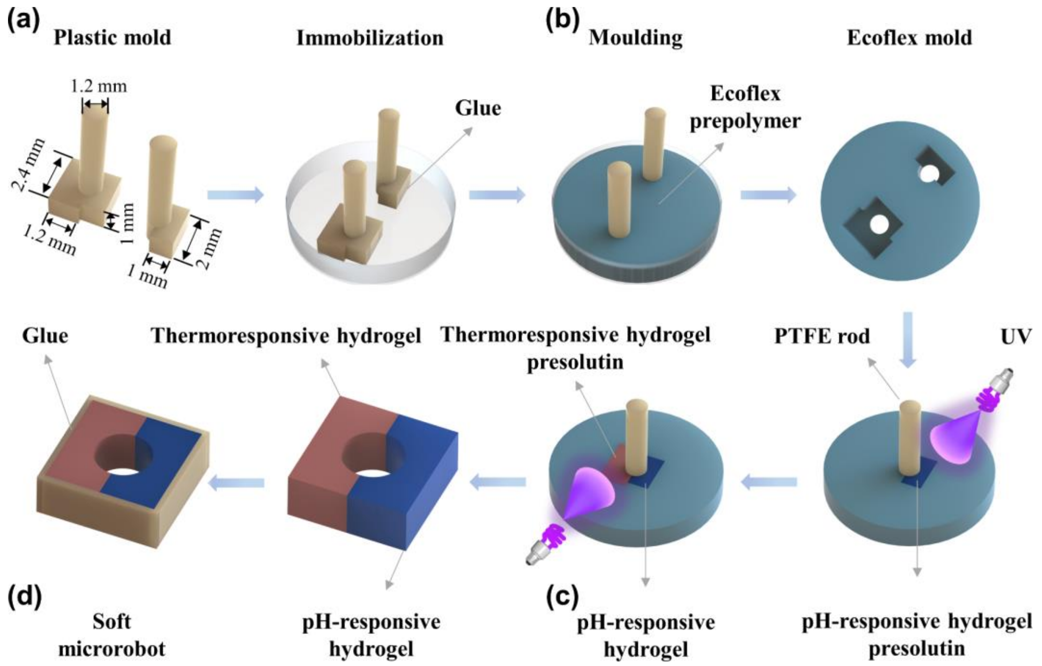

Figure S2. The preparation of soft microrobots with AND and OR gate.

Fabricating the AND-OR connected gate.

The soft microrobot with AND-OR connected gate was prepared using the same hydrogels and similar micromolding technique as used in the AND gate. The precursor solution for the salt-responsive hydrogel was composed of AMPS 0.2 g, AAm 1 g, MBAA 9 mg, V50 10 mg, DI water 2.5 g. After obtained the hydrogel with AND gate, the pH-sensitive hydrogel section was cut in half and re-transferred into the Ecoflex mold of AND gate. Then, the precursor solution of the salt-responsive hydrogel was introduced into the Ecoflex mold and polymerized under UV irradiation for 20 min. Next, the hydrogel was immersed into water overnight to remove the unreacted monomer and initiator. Finally, the four sides of the hydrogel were fixed with glue to obtain the final soft microrobot with the AND-OR gate (the thickness of glue: 200 μm), as shown in **Figure** S3.

Fabricating the OR-OR connected gate.

The soft microrobot with OR-OR connected gate was prepared using the same hydrogels and similar micromolding technique as used in the OR gate (the thickness of glue: 200 μm). The construct process of the soft robot with OR-OR connected gate was consistent with the AND-OR connected gate.

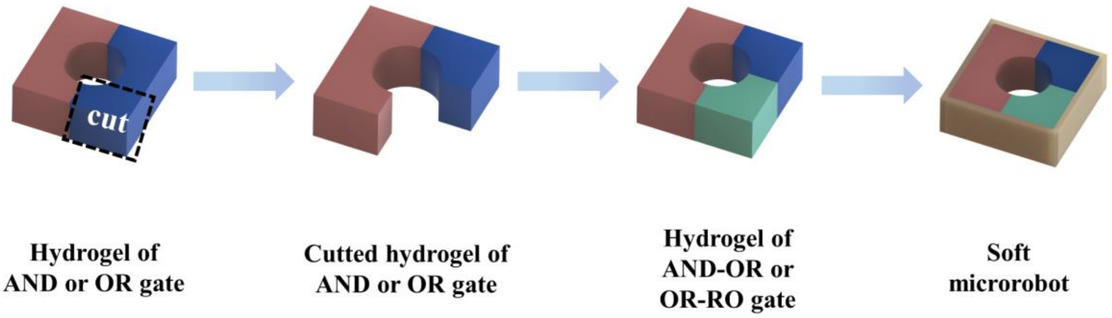

Figure S3. The preparation of soft microrobots with AND-OR and OR-OR connected gate.

Reversibility of soft microrobots with YES gate.

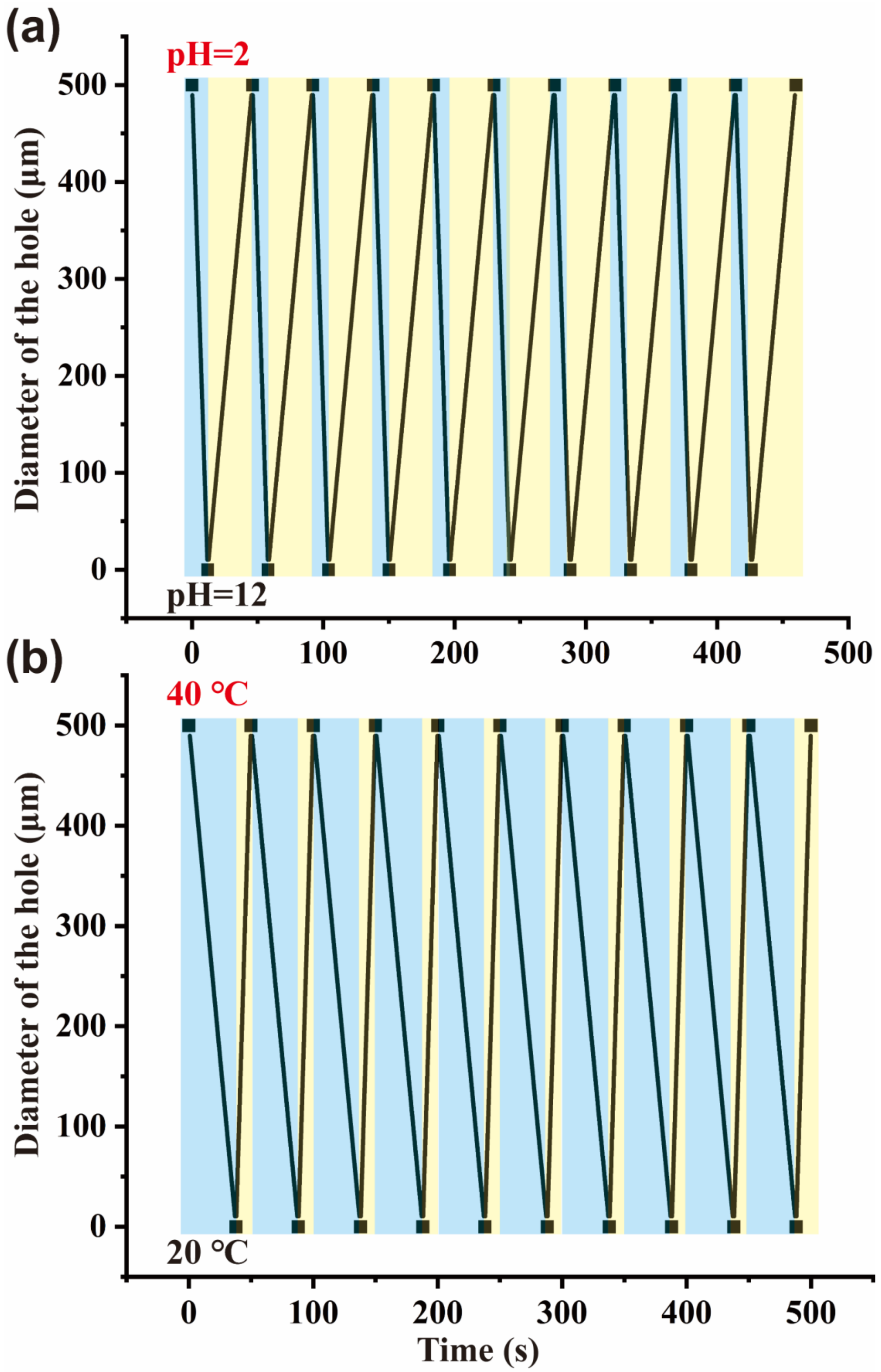

Figure S4. Reversibility of soft microrobots with YES gate. a) soft microrobots with YES gate actuated by pH; b) soft microrobots with YES gate actuated by temperature.

Fabricating the microrobot using dual-responsive hydrogel.

The soft microrobot with dual-responsive hydrogel was prepared using the similar micromolding technique. The precursor solution for the dual-responsive hydrogel layer was composed of AMPS 0.174 g, sodium hydroxide 0.03 g, HEMA 2.51 g, AAc 0.75 g, EGDA 0.03 g, V50 0.1024 g, deionized water 4.615 g. For varying the thickness of responsive layer and passive layer, appropriate Ecoflex mold were selected. First, a kind of plastic mold (2 mm ×2 mm × 1 mm) with a rod in the center (diameter: 1 mm) were fabricated using a printed plastic mold by a 3D printer as shown in **Figure** S5a. The Ecoflex mold was obtained by molding as shown in Figure S5b. The precursor solution for the dual-responsive hydrogel was introduced into the Ecoflex mold with through injector. This mixture was polymerized under UV irradiation for 20 min. Next, the hydrogel was immersed into water overnight to remove the unreacted monomer and initiator. After that, the four sides of the hydrogel were fixed with glue to obtain the final soft robot as shown in Figure S5d (the thickness of glue : 200 μm).

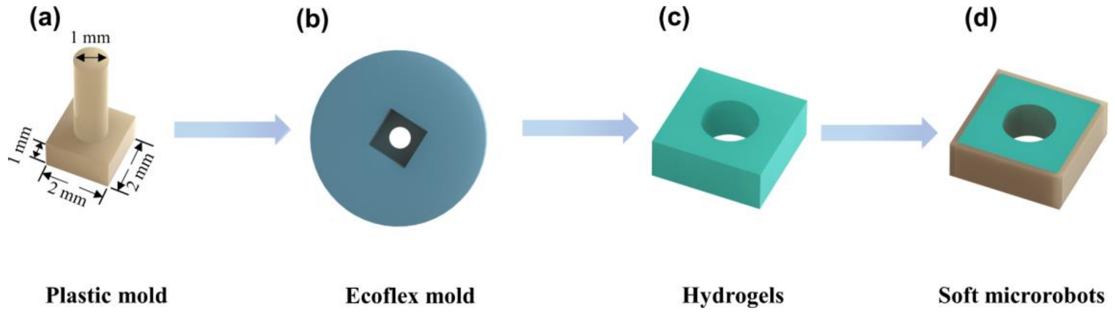

Figure S5. The preparation of soft robots with dual-responsive hydrogel.

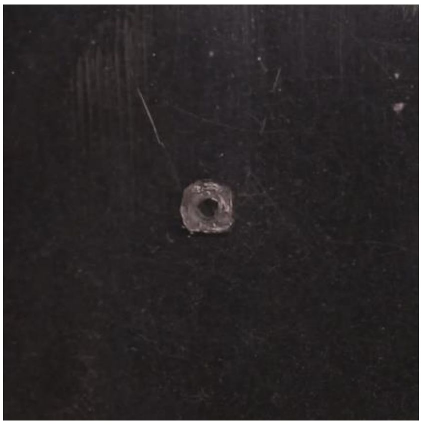

Figure S6. The process of pH and salt dual-responsive hydrogel performing OR gate in an aqueous solution of pH=8.

Fabricating the magnetic actuated soft robot with OR gate.

The magnetic actuated soft robot was prepared by incorporating magnetic nanoparticles, 5wt% Fe_3O_4 nanoparticle, into the precursor solution. The preparation procedure and the hydrogel composition were the same as used in the dual-responsive hydrogel gate (the detailed size of the soft robot was the same as shown in Fig.S1). The H-shaped chamber was prepared by 3D printer. The inner surface of the chamber was coated with agarose gel to decrease the friction between the soft microrobot and the bottom surface of the chamber.

Expectation

Although a series of promising soft microrobot have been developed, the simplification of functions still hinders their further development. Therefore, adding different functional nano fillers and to designing the physical or chemical properties of materials can endow the soft microrobot more functionality, such as conductivity, scalability repeatability, self-healing and degradation.
